# Supplementary material for: Comparison of the data mining and machine learning algorithms for predicting the final body weight for Romane sheep breed
Source: PLoS One. 2023 Aug 3;18(8):e0289348. doi: 10.1371/journal.pone.0289348 (PMC10399827; doi:10.1371/journal.pone.0289348)
Supplement: Attachment — Submitted filename: PONE-D-23-13209.pdf [file pone.0289348.s001.pdf]

PLOS ONE

Comparison of the Data Mining and Machine Learning Algorithms for Predicting the Final Body Weight for Romane Sheep Breed

--Manuscript Draft--

**Manuscript Number:**	PONE-D-23-13209
**Article Type:**	Research Article
**Full Title:**	Comparison of the Data Mining and Machine Learning Algorithms for Predicting the Final Body Weight for Romane Sheep Breed
**Short Title:**	Comparison of the Data Mining and Machine Learning Algorithms
**Corresponding Author:**	Cem Tırınk<br>Igdir University: Igdir Universitesi<br>Igdir, Iğdır TURKEY
**Keywords:**	CART; MARS; Support vector regression; Romane; Sheep
**Abstract:**	The current study aimed to predict final body weight (weight of fourth months of age to select the future reproducers) by using birth weight, birth type, sex, suckling weight, age of suckling weight, weaning weight, age of weaning weight, and age of final weight for the Romane sheep breed. For this aim, classification and regression tree (CART), multivariate adaptive regression splines (MARS), and support vector machine regression (SVR) algorithms were used for training (80%) and test (20%) sets. To predict final body weight, 393 Romane sheep (238 female and 155 male animals) were used with different data mining and machine learning algorithms. The best pre-diction model was obtained by CART model, both training and test set. Constructed CART models indicated that sex, suckling weight, weaning weight, age of weaning weight, and age of final weight could be used as an indirect selection measure to get an elite sheep flock on the final body weight of Romane sheep. If genetically established, the Romane sheep whose sex is female, age of final weight is over 142 days, and weaning weight is over 28 kg could be chosen for affording genetic improvement in final body weight. In conclusion, the usage of CART procedure may be worthy of reflection for identifying breed standards and choosing superior sheep for meat yield in France.
**Order of Authors:**	Cem Tırınk
	Hasan Önder
	Dominique Francois
	Didier Marcon
	Uğur Şen
	Kymbat Shaikenova
	Karlygash Omarova
	Thobela Louis Tyasi
**Additional Information:**	
**Question**	**Response**
**Financial Disclosure**<br><br>Enter a financial disclosure statement that describes the sources of funding for the work included in this submission. Review the submission guidelines for detailed requirements. View published research articles from *PLOS ONE* for specific examples.	There is no financial disclosure.

Powered by Editorial Manager® and ProduXion Manager® from Aries Systems Corporation

This statement is required for submission and **will appear in the published article** if the submission is accepted. Please make sure it is accurate.<br><br>**Unfunded studies**<br>Enter: *The author(s) received no specific funding for this work.*<br><br>**Funded studies**<br>Enter a statement with the following details:<br>• Initials of the authors who received each award<br>• Grant numbers awarded to each author<br>• The full name of each funder<br>• URL of each funder website<br>• Did the sponsors or funders play any role in the study design, data collection and analysis, decision to publish, or preparation of the manuscript?<br>• **NO** - Include this sentence at the end of your statement: *The funders had no role in study design, data collection and analysis, decision to publish, or preparation of the manuscript.*<br>• **YES** - Specify the role(s) played.<br><br>* typeset	
**Competing Interests**<br><br>Use the instructions below to enter a competing interest statement for this submission. On behalf of all authors, disclose any competing interests that could be perceived to bias this work—acknowledging all financial support and any other relevant financial or non-financial competing interests.<br><br>This statement is required for submission and **will appear in the published article** if the submission is accepted. Please make sure it is accurate and that any funding sources listed in your Funding Information later in the submission form are also declared in your Financial Disclosure statement.<br><br>View published research articles from *PLOS ONE* for specific examples.	There is no competing interest.

**NO authors have competing interests**<br><br>Enter: *The authors have declared that no competing interests exist.*<br><br>**Authors with competing interests**<br><br>Enter competing interest details beginning with this statement:<br><br>*I have read the journal's policy and the authors of this manuscript have the following competing interests: [insert competing interests here]*<br><br>* typeset	
**Ethics Statement**<br><br>Enter an ethics statement for this submission. This statement is required if the study involved:<br><br>• Human participants<br>• Human specimens or tissue<br>• Vertebrate animals or cephalopods<br>• Vertebrate embryos or tissues<br>• Field research<br><br>Write "N/A" if the submission does not require an ethics statement.<br><br>General guidance is provided below. Consult the submission guidelines for detailed instructions. **Make sure that all information entered here is included in the Methods section of the manuscript.**	There is no need to take ethical approval because there is no clinical application on the animals.

**Format for specific study types**<br><br>**Human Subject Research (involving human participants and/or tissue)**<br>• Give the name of the institutional review board or ethics committee that approved the study<br>• Include the approval number and/or a statement indicating approval of this research<br>• Indicate the form of consent obtained (written/oral) or the reason that consent was not obtained (e.g. the data were analyzed anonymously)<br><br>**Animal Research (involving vertebrate animals, embryos or tissues)**<br>• Provide the name of the Institutional Animal Care and Use Committee (IACUC) or other relevant ethics board that reviewed the study protocol, and indicate whether they approved this research or granted a formal waiver of ethical approval<br>• Include an approval number if one was obtained<br>• If the study involved *non-human primates*, add *additional details* about animal welfare and steps taken to ameliorate suffering<br>• If anesthesia, euthanasia, or any kind of animal sacrifice is part of the study, include briefly which substances and/or methods were applied<br><br>**Field Research**<br><br>Include the following details if this study involves the collection of plant, animal, or other materials from a natural setting:<br>• Field permit number<br>• Name of the institution or relevant body that granted permission	
**Data Availability**<br><br>Authors are required to make all data underlying the findings described fully available, without restriction, and from the time of publication. PLOS allows rare exceptions to address legal and ethical concerns. See the PLOS Data Policy and FAQ for detailed information.	No - some restrictions will apply

Powered by Editorial Manager® and ProduXion Manager® from Aries Systems Corporation

A Data Availability Statement describing where the data can be found is required at submission. Your answers to this question constitute the Data Availability Statement and **will be published in the article**, if accepted.<br><br>**Important:** Stating 'data available on request from the author' is not sufficient. If your data are only available upon request, select 'No' for the first question and explain your exceptional situation in the text box.<br><br>Do the authors confirm that all data underlying the findings described in their manuscript are fully available without restriction?	
**Describe where the data may be found in full sentences. If you are copying our sample text, replace any instances of XXX with the appropriate details.**<br><br>• If the data are **held or will be held in a public repository**, include URLs, accession numbers or DOIs. If this information will only be available after acceptance, indicate this by ticking the box below. For example: *All XXX files are available from the XXX database (accession number(s) XXX, XXX.).*<br>• If the data are all contained **within the manuscript and/or Supporting Information files**, enter the following: *All relevant data are within the manuscript and its Supporting Information files.*<br>• If neither of these applies but you are able to provide **details of access elsewhere**, with or without limitations, please do so. For example:<br><br>*Data cannot be shared publicly because of [XXX]. Data are available from the XXX Institutional Data Access / Ethics Committee (contact via XXX) for researchers who meet the criteria for access to confidential data.*<br><br>*The data underlying the results presented in the study are available from (include the name of the third party*	Not applicable.

and contact information or URL).<br>• This text is appropriate if the data are owned by a third party and authors do not have permission to share the data.<br><br>* typeset	
Additional data availability information:	

Manuscript
Click here to access/download;Manuscript;Manuscript_PlosOne.docx

Comparison of the Data Mining and Machine Learning Algorithms for Predicting the Final Body Weight for Romane Sheep Breed

Cem Tırınk[1,*], Hasan Önder[2], Dominique Francois[3], Didier Marcon[4], Uğur Şen[5], Kymbat Shaikenova[6], Karlygash Omarova[6], Thobela Louis Tyasi[7]

[1] Department of Animal Science, Faculty of Agriculture, Igdir University, 76000, Iğdır, Türkiye

[2] Department of Animal Science, Faculty of Agriculture, Ondokuz Mayis University, 55139, Samsun, Türkiye

[3] GenPhySE, INRA, INPT, INP - ENVT, Université de Toulouse, Castanet Tolosan, France

[4] INRAE, UE P3R, F18390-Osmoy, France

[5] Department of Agricultural Biotechnology, Faculty of Agriculture, Ondokuz Mayis University, Samsun 55139, Türkiye

[6] Saken Seifullin Kazakh Agrotechnical University, Faculty of Veterinary and Animal Husbandry Technology, Department of Technology and Processing of Livestock Production, Z11F9K5, Astana, Kazakhstan

[7] School of Agricultural and Environmental Sciences, Department of Agricultural Economics and Animal Production, University of Limpopo, Private Bag X1106, Sovenga 0727, Limpopo, South Africa

[*]Corresponding author's e-mail: cem.tirink@gmail.com

Abstract

The current study aimed to predict final body weight (weight of fourth months of age to select the future reproducers) by using birth weight, birth type, sex, suckling weight, age of suckling weight, weaning weight, age of weaning weight, and age of final weight for the Romane sheep breed. For this aim, classification and regression tree (CART), multivariate adaptive regression splines (MARS), and support vector machine regression (SVR) algorithms were used for training (80%) and test (20%) sets. To predict final body weight, 393 Romane sheep (238 female and 155 male animals) were used with different data mining and machine learning

algorithms. The best pre-diction model was obtained by CART model, both training and test set. Constructed CART models indicated that sex, suckling weight, weaning weight, age of weaning weight, and age of final weight could be used as an indirect selection measure to get an elite sheep flock on the final body weight of Romane sheep. If genetically established, the Romane sheep whose sex is female, age of final weight is over 142 days, and weaning weight is over 28 kg could be chosen for affording genetic improvement in final body weight. In conclusion, the usage of CART procedure may be worthy of reflection for identifying breed standards and choosing superior sheep for meat yield in France.

Keywords: CART; MARS; Support vector regression; Romane; Sheep

Introduction

Sheep, ~~one of the animal species,~~ is one of the first domesticated animals within the scope of archaeological and genetic studies [1,2]. As a result of this domestication process, it has led to an improvement in sheep breeding over time. Sheep breeding is a multi-purpose small ruminant animal that is very valuable not only for the development of healthy civilizations but also for obtaining animal products such as milk, meat, and fleece, especially in the development processes of rural economies [3-6]. According to this im-portance for human being, researchers aimed sheep farms to reach a more profitable, innovative, and sustainable [7]. According to the Food and Agriculture Organization (FAO), 5.5 million heads of sheep are adapted to several regions in France [8]. Principal sheep breeds include Berrichon du Cher, Blanc du Massif Central, Charollais, Roussin de La Hague, Ile de France, Préalpes du Sud, Lacaune and Romane in France [9-11]. Many sheep breeds have adapted to the conditions of the region, as well as herd management is of great importance for sustainable sheep breeding [12].

Knowing the live weights of the animals in the herd in sheep breeding is essential in determining the breeding strategy to be applied in the herd and herd management. In this context, it will provide convenience in calculating the optimum feed amount per sheep, determining the drug doses, marketing price more reliably, and optimum slaughter time of the animals [13-15].

In the literature, there are many studies in which multivariate statistical methods are used together with biometric measurements to create a characterization for breeds [16-19]. In multivariate statistical methods, conditions such as linearity, constant variance, normality, and multicollinearity should be sought within the scope of the regression analysis assumptions [20-23]. ~~Also~~ However, various data mining and machine learning algorithms do not need these assumptions

[24,25]. In this context, many studies within the scope of multivariate statistical methods have been carried out on different breeds and species, in which characteristics such as birth weight, live weight, weaning weight, and final weight are tried to be estimated [26-28]. The main purpose of trying to predict such different traits is that these traits vary from species to species and breed to breed in the same species. Expressing these differences will provide convenience in herd management issues such as selection practices and breed characterization.

This study aimed to estimate the optimum final weight (weight of fourth months of age to select the future reproducers (rams and ewes)) from sex, birth weight, suckling weight, age of suckling weight, weaning weight, age of weaning weight, and age of final weight. For this aim, we used and compared three algorithms such as CART, MARS and SVR algorithms.

Materials and methods

To compare the algorithms, the data were provided from INRA on Romane sheep breed. Romane sheep (Berrichon du Cher by Romanov crossbred) was developed to increase the productivity of the French sheep herd. INRA created the synthetic strain INRA 401 by crossing the Berrichon du Cher breed (good butchering skills but not very prolific, not very maternal, white fleece) with the Romanov breed (very prolific, maternal, but low butchering skills and coloured fleece). The line obtained was named Romane breed in 2006. It has an average litter size of 2 lambs per birth (on 3-year-old ewes), excellent fertility in the off-season, and good viability of the lambs at birth [29]. Housing conditions during the suckling period: ewes and lambs in the barn, ewes are fed with a total mix ration composed of wrapped forage bales and concentrates, and lambs are fed ad-libitum with concentrates. This study used data from 393 animals (155 male and 238 female) born in August, September and October 2021. Sex, birth weight, suckling weight, age of suckling weight (about 30 days), weaning weight, age of weaning weight, and age of final weight were used to estimate the final weight that weight of fourth months of age to select the future reproducers (rams and ewes), still young and not mature adults.

Descriptive statistics of explanatory and response variables were given according to each sex. The comparison of each variable was compared by using the paired sample t-test ($p<0.05$). (??? Paired t-test cannot be used to compare variables. If you want to compare genders by the variables than you must use independent samples t-test, not paired t-test.)

The CART (Classification and Regression Tree) algorithm, one of the tree algorithms utilized to predict body weight, was first proposed by Breiman et al. [30]. With the CART algorithm, a binary split tree structure created by splitting a variable homogeneously includes the two sub-

nodes. In the CART algorithm, the process begins from the root node, including the initial data set, and continues until many homogeneous sub-nodes are gotten, which will supply the minimum error variance.

Multivariate Adaptive Regression Splines (MARS) algorithm was proposed by Friedman [31]. The MARS algorithm is a non-parametric regression procedure that facilitates a more influential description of nonlinear and interaction effects between response and explanatory variables. There is no need for the MARS algorithm assumptions as in linear regression [27,32-34].

The MARS algorithm generates fundamental functions according to the stepwise procedure, considering all possible interaction effects between contestant knots and explanatory variables. The algorithm includes two different stages such as forward and backward pass stages, respectively [35]. The first process of the algorithm is the forward pass process. In this process, the algorithm begins with an intercept in the initial pattern, and to improve the model, iteratively contains the initial patterns coupled with the least training error. The forward pass process typically products an over-fitted pattern that achieves extreme complexity [27,31]. The model built from the forward pass process fits predominantly worthy. Nonetheless, its generalization capability can be frail for the data set before the undecided-built model, which calls for an overfitting difficulty. The initial patterns that specify the smallest amount of the prediction model are abolished in the backward pass process, and this process is hand-me-down in the resolution to this problem [18,35,36]. MARS is a statistically significant tool that can capture the relationship between dependent and independent variables [37, 38]. The equation for MARS procedure utilized to predict body weight from explanatory variables is below.

$$\hat{y} = \beta_0 + \sum_{m=1}^{M} \beta_m \prod_{k=1}^{K_m} h_{km}(X_{v(k,m)}) \quad (1)$$

where, $\hat{y}$ is the predicted value of the outcome variable (BW), β_0 is an intercept of the model, β_m is the basis functions coefficient, K_m is the parameter that limits the interaction order, h_{km} $(X_{v(k,m)})$ term is called the basis function and v(k,m) is an index of the explanatory variable in the mth component of the kth product. The basic functions that reduce the performance of the model obtained after the forward and backward pass stages are eliminated due to the generalized cross-validation error (GCV). The formula for GCV is given below [27,36,39]:

$$\mathrm{GCV}(\lambda) = \frac{\sum_{i=1}^{n}(y_i - \hat{y}_i)^2}{\left[1 - \frac{M(\lambda)}{n}\right]^2} \quad (2)$$

where: n is the size of training set, y_i is the observed value for the response variable (BW), ($\hat{y}_i$) is the estimated value for the response variable (BW), M(λ) is called the penalty term for the complexity for the model that includes the λ terms.

At the initiation of the MARS procedure, the relationship between the explanatory variables, called as multicollinearity, were tested and it was determined that there was no multicollinearity problem between the explanatory variables. To predict BW utilizing in the training set. The cross-validation procedure was helped to select the greatest MARS model among 72 MARS model (degree = 1:2 and nprune = 2:38). In addition, for training data set, ten-fold cross-validation technique was utilized for optimal MARS model.

A significant twig of the support vector machine (SVM), which is a machine learning algorithm, is the support vector regression (SVR) algorithm [40]. Here, to struggle with classification is named support vector classification (SVC), while to struggle with modeling and prediction is named SVR [33,41-43]. Although SVR is also a supervised learning method, the prediction performance obtained from SVR varies depending on the training and test set [33,44].

The main goal in the linear SVR model is to define a function of f(x) which can have the maximum deviation (ε) from the training set. Training set points are built into the boundary between −ε to +ε [44]. However, in most studies, it cannot be shown within the scope of linear properties. Therefore, when using nonlinear SVR, the input data is matched to a higher dimensional Hilbert space (H) so that the regression line can be linear [40].

The hyperplane of the nonlinear SVR to be obtained is as follows;

$$\hat{y} = \langle w, \phi(x) \rangle + b \quad (3)$$

In this equation, weight vector is defined by w, non-linear kernel functions are defined by ϕ(x), indicates vector inner product is defined by ⟨.,.⟩ and the term of b is a bias term. There are many nonlinear kernel functions. In the current study, gaussian radial basis kernel function were used.

In comparison of the model performances, the following goodness of fit criteria were used [27,28,33,36,45]:

1. Root-mean-square error (RMSE):

$$\mathrm{RMSE} = \sqrt{\frac{1}{n}\sum_{i=1}^{n}\left(y_i - y_{ip}\right)^2} \quad (4)$$

2. Akaike information criterion (AIC):

$$\begin{cases} AIC = n.ln\left[\frac{1}{n}\sum_{i=1}^{n}\left(y_i - y_{ip}\right)^2\right] + 2k, & \text{if } n/k > 40 \\ AIC_c = AIC + \frac{2k(k+1)}{n-k-1} & otherwise \end{cases} \quad (5)$$

3. Standard deviation ratio (SD_{ratio}):

$$SD_{ratio} = \frac{S_m}{S_d} \quad (6)$$

4. Global relative approximation error (RAE):

$$\mathrm{RAE} = \sqrt{\frac{\sum_{i=1}^{n}\left(y_i - y_{ip}\right)^2}{\sum_{i=1}^{n} {y_i}^2}} \quad (7)$$

6. Mean absolute percentage error (MAPE):

$$\mathrm{MAPE} = \frac{1}{n}\sum_{i=1}^{n}\left|\frac{y_i - y_{ip}}{y_i}\right| * 100 \quad (8)$$

7. Pearson correlation coefficient (r):

$$r = \frac{\sum(x_i - \bar{x})(y_i - \bar{y})}{\sqrt{\sum(x_i - \bar{x})^2 \sum(y_i - \bar{y})^2}} \quad (9)$$

8. Performance Index (PI):

$$\mathrm{PI} = \frac{\mathrm{rRMSE}}{1+r} \quad (10)$$

where, n is the size of training data set, k is called the number of parameters for the model, y_i is the real value of the response variable (BW), $\hat{y}_i$ is the predicted value for response variable (BW), s_d is the standard deviation for the response variable (BW), s_m is the standard deviation for optimum model's errors [33].

RMSE, SD_{ratio}, CV, PI, RAE, MAPE and AIC, r and R^2 criteria were used to compare the performance of the model. For this aim, it needs to determine best model for smallest RMSE, SD_{ratio}, CV, PI, RAE, MAPE and AIC values for train and test set and also, the highest r, R^2 value for all algorithms [46].

All statistical evaluation was performed using R software [47]. To required knowledge of the data, descriptive statistics were utilized. The descriptive statistics for explanatory and response variables were predicted by using "psych" package in R environment [48]. The "caret" packages in the R software were used to perform analyze of the CART and MARS algorithms [49]. Also, support vector regression algorithm was performed by using "e1071" package in R

software [50]. To display the performances of the constructed all models, the "ehaGoF" package was utilized [51].

Results

Descriptive statistics for all variables by sex (male and female) factor were expressed as mean ± standard error and are given in Table 1.

Table 1. Descriptive statistics for the whole characteristics

Traits	Male	Female
Birth weight	4.39 ± 0.06	4.39 ± 0.05
Suckling weight	12.66 ± 0.19	12.59 ± 0.14
Age of suckling weight	30.74 ± 0.19	31.17 ± 0.13
Weaning weight	26.29 ± 0.38	24.78 ± 0.27
Age of weaning weight	64.30 ± 0.24	64.66 ± 0.21
Final weight	42.16 ± 0.37[a]	35.75 ± 0.34[b]
Age of final weight	109.61 ± 0.35	105.95 ± 0.83

a,b: different letters in same row shows the statistical difference ($p<0,05$). Is this letter given according to paired t-test results ???? If so, the analysis should be done from scratch according to the t-test.

Pearson's correlation coefficients for defining the association with response and explanatory variables except for age of suckling weight, age of weaning weight, and age of final weight. Final body weight had a small correlation coefficient with the birth weight, suckling weight, weaning weight with the coefficients of 0.29, 0.42, 0.52, respectively. Among the explanatory variables, relatively high correlation was determined only between weaning weight and suckling weight. The other correlation coefficients were low or moderate and can be interpreted as having no relationship.

Table 2. Descriptive statistics for the whole characteristics.

	Birth weight	Suckling weight	Weaning weight	Final body weight
Birth weight	1			
Suckling weight	0.68	1		
Weaning weight	0.53	0.79	1	
Final body weight	0.29	0.42	0.52	1

Figure 1 points out the constructed CART regression tree diagram in estimation of final weight from explanatory variables and sex (1=male, 2=female) factor.

≪Figure 1. Constructed CART regression tree diagram.≫

The final weight of the Romane sheep breed was divided into two groups within the scope of sex. On the left side of the diagram, if the sex was female, the mean of the final body weight was determined as approximately 36 kg. The female Romane sheep were divided into two groups within the scope of weaning weight; weaning weight <23 kg and weaning weight ≥23

kg. If the weaning weight was ≥23 kg, the tree was divided into the age of final weight<106 days with the mean of 37 kg. In addition, if the age of the final weight was above 106 days, the final weight was divided into two groups as for weaning weight was over and under 27 kg. According to Figure 1, if the sex was female, weaning weight ≥23 kg, age of final weight≥106 days, and weaning weight≥27 kg, the predicted final weight was determined as 45 kg. On the right side of the diagram, if the sex was male, the mean of the final body weight was determined as approximately 42 kg. The male Romane sheep were divided into two groups for the age of final weight at 142 days. If the age of final weight is under 142 days, the final body weight was divided into for suckling weight with the coefficient of 18 kg. In addition, if the sex was male, the age of final weight was over 142 days, weaning weight over 28 kg, the final body weight was determined as 64 kg.

Table 3 gives the results of the CART algorithm depending on the cross-validation procedure. The constructed CART regression tree algorithm made 12 terminal knots (size of regression tree) with relative error (0.148) and the cross-validation error (0.312) which means that coefficient of determination (R^2) and cross-validation R^2 were close to each other with 0.852 (1–0.148) and 0.688 (1–0.312), respectively (Table 3). The relative error can estimate the R^2 and cross-validation error can be defined as the mean of the cross-validated prediction errors and cross-validation R^2 is calculated by 1–relative error. Cross-validation standard deviation can be defined the standard deviation of the cross-validated prediction errors (Table 3).

Table 3. Results of CART algorithm based on the cross-validation technique.

	Complexity parameter	Number of splits	Relative error	Cross-validation error	Cross-validation standard deviation
1	0.294	0.000	1.000	1.014	0.138
2	0.177	1.000	0.706	0.759	0.103
3	0.138	2.000	0.530	0.675	0.096
4	0.067	3.000	0.391	0.451	0.050
5	0.046	4.000	0.325	0.399	0.048
6	0.042	5.000	0.279	0.384	0.046
7	0.028	6.000	0.237	0.318	0.042
8	0.017	7.000	0.208	0.320	0.045
9	0.015	8.000	0.191	0.322	0.045
10	0.015	9.000	0.176	0.323	0.046

11	0.013	10.000	0.161	0.311	0.045
12	0.010	11.000	0.148	0.312	0.045

Table 4 shows that the final body weight can be explained with the eight basis functions in the MARS prediction model. According to Table 4, the first term of the MARS prediction model was an intercept that had a coefficient of 36.770. In the second term, the case where the sex is female for a negative coefficient of -4.369. The third term (Suckling weight-12.8) had a cutpoint of 12.8 kg with a coefficient of 0.814. The fourth term was the suckling weight age, with a cutpoint of 35 with a coefficient of 0.415.. For the age of suckling weight is 35 days, the effect of term four on final body weight was unavailable. The fifth and sixth terms were for weaning weight, with cutpoint 17.4 and 24.4 with a coefficient of 1.0817 and -0.616, respectively. When weaning weight was 17.4 and 24.4 kg or lighter, no contribution of the weaning weight to the final body weight was found. For the weaning weight lighter than 17.4 kg, term 5 was useless, and then only the term 6 (Weaning weight-24.4) contributed to the final body weight. The same procedure was for the term of 6. If the weaning weight was lighter than 24.4 kg, the term of 6 was useless. The seventh and the last term of the MARS prediction model were for the age of final weight with a cutpoint of 133 days.

Within the scope of estimating final body weight, the obtained best prediction MARS model allows breeders to get into more precise decisions considering herd management, such as the appropriate feed amount, medicinal drug doses for Romane sheep breed, and establishing the selling value of each sheep.

Table 4. The obtained MARS model for Romane sheep

Explanatory variables	Coefficients
Intercept	36.770
Sex-Female	-4.369
h (Suckling weight-12.8)	0.814
h (35-Age of suckling wight)	0.415
h (Weaning weight-17.4)	1.0817
h (Weaning weight-24.4)	-0.616
h (133-Age of final weight)	-0.222
h (Age of final weight-133)	0.324

At the start of the SVR algorithm was instructed to the training set. Later performing the training processes, the SVR was assessed to predict the final body weight for Romane sheep breed. In the current study, the gaussian radial basis kernel function was used for estimating the final body weight of the Romane sheep breed. The consistency of the model based on the selection parameters such as cost (C), gamma and epsilon. The current study determined cost,

gamma, and epsilon values as 1, 0.083, and 0.1, respectively. These parameters were verified for several values, and analysis was affected for C and epsilon values. So, it would provide the greatest trustworthy model. To determine which variable is more effective, sensitivity analysis was used. The sensitivity analysis was calculated to estimate the relative importance values of the affected explanatory variables on final body weight. In Figure 2, the sensitivity analysis results were given.

≪Figure 2. Sensitivity analysis for support vector regression algorithm≫

According to Figure 2, the age of final weight was a more effective variable on final body weight for support vector regression algorithms. The second effective variable was weaning weight. Apart from these variables, variables sex (male and female), suckling weight, age of weaning weight, and birth weight were also important in determining the final body weight. The least effective variables in determining the final body weight were the age of suckling weight, birth type (2, 3, and 4), and number of co-suckled lambs.

For comparing all models, the goodness of fit criteria was used. The model performance results for CART, MARS, and SVR algorithms, based on the goodness-of-fit criteria, were provided in Table 5. Table 5 shows the finest prognostic model was realized for CART algorithm. The CART algorithm had the smallest RMSE, SD_{ratio}, CV, PI, RAE, MAPE, and AIC values for the train and test set. Also, the highest R^2 value was determined for the CART algorithm.

Table 5. Goodness-of-fit criteria for CART, MARS and SVR algorithms

Goodness-of-fit criteria	CART		MARS		SVR	
	Train	Test	Train	Test	Train	Test
RMSE	2.264	2.586	2.466	2.867	2.404	2.898
SD_{ratio}	0.385	0.434	0.419	0.483	0.409	0.489
CV	5.920	6.790	6.450	7.550	6.280	7.640
r	0.923	0.902	0.908	0.876	0.924	0.882
PI	3.075	3.563	3.375	4.004	3.263	4.034
RAE	0.003	0.004	0.004	0.006	0.004	0.006
MAPE	4.856	5.596	5.022	5.484	4.162	5.773
R^2	0.852	0.810	0.824	0.766	0.833	0.761
AIC	516.504	146.321	586.353	178.198	554.251	163.863

In addition, Pearson's correlation coefficient was determined to be 0.923 and 0.902 for the original data and predicted data for training and test sets, respectively. Furthermore, coefficient of variation (CV) values was under 30%. It means that the results of this study were reliable.

Discussion

The CART algorithm is helpful for identifying the most important factors affecting body weight and provides a way to rank these factors in order of importance. The CART algorithm can help breeders identify the best animals for increasing the aimed yield or trait properties. Additionally, CART can be used to identify patterns in sheep body weight data, which can be used to predict future weights and adjust breeding practices accordingly. In addition, the number of studies comparing these algorithms is relatively low. In addition, it should be noted that algorithms may differ from species to species and even from breed to breed in the same species.

Alonso et al. [43] utilized the SVR algorithm to predict the carcass weight in Asturiana de los Valles beef. To predict the carcass weight, the study was made using 390 measurements from 144 animals. According to the study's results, the ideal carcass weight prediction was obtained 150 days before slaughter time. They found MAPE values as 4.12 and 4.91 for train and test, respectively. These values were smaller than our findings. The achievement of their study may be caused by a sample size that was greater than ours.

Celik et al. [3] aimed to compare CART, CHAID, Exhaustive CHAID, MARS, MLP, and RBF for Mengali rams. The supreme prediction model was described as the CART algorithm in the scope of the model comparison criteria such as R^2, RMSE and SD_{ratio}. The only matching algorithm was CART and MARS, which we also utilized in our study. The CART algorithm was found to be more reliable than the MARS algorithm in the work of Celik et al. [3], which supported our current study results.

Iqbal et al. [52] was targeted to compare the model performances for regression tree algorithms, gradient boosting machine, random forests, and SVR algorithms. For this aim, Iqbal et al. [52] was used the data obtained from Beetal goats. To predict the body weight, they used explanatory variables such as sex, body length, shank circumference, neck length, head girth, rump height, and belly sprung were evaluated. This study selected model comparison criteria such as Pearson's correlation, R^2, RMSE, MAPE, and MAE. According to the results of Iqbal et al. [52], the gradient boosting machine was stated to be the greatest model for estimating the

body weight of Beetal goats. However, the random forest regression algorithm was determined to be the second-best algorithm. Even if the SVR algorithm does not give the best results, even the data structure impacts on the prediction performances.

Marco et al. [53] aimed to try to determine AdaBoost ensemble learning method and RFR for different data sets. Several machine learning techniques, such as CART, kNN, MLP, SVR, and RFR were used for different data sets. According to the results of most of the datasets applied in the study, they stated that it was a reliable and successful algorithm for RFR. However they indicated that the CART algorithm was also a useable algorithm, even if the CART algorithm wasn't very sensitive to parameter tuning when RFR was so sensitive to parameter tuning, which results in stable prediction performance.

Tırınk [54] aimed to compare various data mining and artificial neural network algorithms such as Bayesian Regularized Neural Network, Multivariate Adaptive Regression Splines, Random Forest Regression and Support Vector Regression algorithms to estimate body weight from body measurements for the Thalli sheep breed. For this aim, 270 female Thalli sheep breeds were used. According to the results, the MARS algorithm was the best prediction model for Thalli sheep breeds inside the Bayesian regularized neural network, random forest regression and support vector regression. These results support our results because the MARS model showed more consistent results than the SVR algorithm.

Tırınk et al. [33] aimed to compare CART, SVR, and RFR data mining and machine learning algorithms to estimate body weight based on body measurements at a different share of Polish Merino in the genotype of crossbreds (share of Suffolk and Polish Merino genotypes). To compare the estimation performances of the evaluated algorithms and determine the best model for estimating body weight, various body measurements and sex and birth type characteristics were assessed. According to test sets results, using random forest regression was recommended instead of using CART and SVR algorithms. The performance of the SVR (R^2=0.714) algorithm was found to be more reliable than CART (R^2=0.578) algorithm by Tırınk et al. [33]. These findings were not compatible with our results. In our study, the CART (R^2=0.810) algorithm was better than the SVR (R^2=0.761) algorithm. But it should be considered that there were such big differences in the point of determination coefficient. They used the data for 344 animals, while our sample size was 393. These sample sizes were not so different; that is the cause of the contrast cannot be. The data structure, such as the distribution, may cause the results to contrast.

Kumar and Kumar-Singh [55] aimed to compare MLR, MARS, SVR, and RFR techniques in hydrological time-series modeling. Their results showed that according to the RMSE and R^2 values, the SVR algorithm was found superior and could be applied to predict weekly pan evaporation values for the Ranichauri site. Their results were comparable only for MARS and SVR algorithms with our results. They suggested the use of SVR, but our results had a different recommendation to use MARS algorithms when compared with the study of Kumar and Kumar-Singh [55]. The coefficient of determination difference was only 0.01, which can be ignored that both MARS and SVR can be interpreted as similar, as in our results.

Komadja et al. [56] aimed to develop a model to predict peak particle velocity (PPV) in opencast mines using CART, MARS, and SVR algorithms. The models were developed using a record of 1001 real blast-induced ground vibrations, with ten corresponding blasting parameters from 34 opencast mines/quarries from India and Benin. The results showed that the MARS model outperformed other models in this study with lower error (RMSE= 0.227) and R^2 of 0.951, followed by SVR (R^2= 0.87), CART (R^2= 0.74), and empirical predictors. Their results completely contrast to our findings that our study showed the CART algorithm was the best. Also, Komadja et al. [56] gave 50 results of previous studies results in a list. Interpretation of the list showed no consensus on the algorithm selection. The contrast in the results may be caused by data type and sample size.

Compared to the abovementioned research, several species and breeds were used for data mining and machine learning algorithms. The extensive variation in previous studies was attributed to the animal age, differences in flock management systems, and differences in statistical methods applied. When our study was compared with other results, it was determined that the models we used according to the selected goodness-of-fit criteria gave similar results. However, recommending several statistical procedures for BW estimation using biometric features is important for both species and breed characterization for meat production industries. It reveals that more studies are needed on this subject.

Conclusion

However, some studies predict body weight for various animal species and breeds. For example, there are studies on some cows. In these studies, genetic and environmental factors that determine the growth rate, age, sex, performance, and feeding condition of the cows are taken into consideration. In some studies, regression and decision tree models have also been used to predict the body weight of cows. Similarly, similar studies have been done for some

other animals as well. In these studies, various models have been used to predict the body weight of various animals. Among the methods included in these models are regression, support vector machines (SVMs), decision trees, k-nearest neighbor algorithms (KNN), and neural networks. However, no study has been found comparing CART, MARS and SVR algorithms that predict final body weight. Thus, further research is needed to compare these algorithms and determine which is the most suitable for predicting body weight in livestock.

The CART, MARS, and SVR algorithms are powerful tools for predicting and analyzing data in animal science as in other fields. CART is a powerful tool for decision-making, MARS is a powerful tool for exploring complex relationships in data, and SVR is a powerful tool for predicting future values. However, all three of these algorithms have their limitations. CART is limited to binary decisions, MARS is limited to linear relationships, and SVR is limited to small datasets. Additionally, all three algorithms require considerable data preparation and can be computationally intensive. As such, animal scientists must be aware of the strengths and weaknesses of these algorithms in order to determine which is best suited for their research.

The body weight of the sheep breeds is a crucial factor to consider when selecting a breed for a certain purpose. It is essential to assess the body weight and size of the breed to ensure that they are suitable for their intended purpose and to ensure that they will not become overweight or underweight due to their diet and lifestyle. By assessing the body weight of different breeds of sheep, one can make an informed decision when selecting the right breed for their needs. By understanding the importance of determining body weight for different sheep breeds, one can select the right breed for their needs and ensure healthy, productive livestock. Multivariate statistical methods are essential for determining body weight in different breeds of sheep. It allows for more accurate estimates of the breed's body weight and size and helps identify any potential problems with the breed. These methods can help improve the livestock's health and productivity and ensure that the right breed is chosen for the intended purpose.

The CART algorithm is a powerful statistical tool that can be used to accurately determine a sheep's body weight. It uses a decision tree to analyze the data and can be used to identify patterns in the data that can be used to predict the body weight of sheep accurately. The CART algorithm is beneficial for breeders who need to accurately predict the weights of their animals for breeding and marketing purposes. CART can also be used to identify any potential diseases or health issues that may affect the body weight of the sheep, allowing breeders to take steps to address any potential issues.

Supporting information

S1 File. Constructed CART regression tree diagram. The obtained CART tree diagram results of the analysis.

(PDF)

S2 File. Sensitivity analysis for support vector regression algorithm. According to the SVR algorithm, it was determined the most effective explanatory variables within the scope of sensitivity analysis.

(PDF)

Author Contributions

Conceptualisation, C.T. and H.Ö.; methodology, C.T., D.F., D.M., H.Ö.; validation, C.T., U.Ş. and H.Ö.; formal analysis, C.T., K.S. and H.Ö.; investigation, C.T. and H.Ö.; resources, C.T., D.F. and D.M.; data collection, D.F. and D.M.; data analysis, H.Ö., C.T. and K.O.; writing—original draft preparation, C.T., K.S., K.O., H.Ö., U.Ş., T.L.T; writing—review and editing, C.T., K.S., K.O., H.Ö., U.Ş., T.L.T; visualisation, C.T. and H.Ö.; supervision, C.T., D.F. and H.Ö.; project administration, D.F., D.M. All authors have read and agreed to the published version of the manuscript.

References

1. Zeder MA. Domestication and early agriculture in the Mediterranean Basin: origins diffusion, and impact. Proc. Natl. Acad. Sci. 2008; 105: 11597-11604.
2. Şirin E. The effect on fertility of using different doses of PMSG in Anatolian merino sheep. BSJ Agric. 2022; 5(3): 278-280.
3. Celik S, Eyduran E, Karadas K, Tariq MM. Comparison of predictive performance of data mining algorithms in predicting body weight in Mengali rams of Pakistan. Braz J Anim Sci.2017; 46(11): 863-872.
4. Erkan Can M, Boğa M. The importance of sheep shelters in cold climate region. BSJ Agric. 2018; 1(1): 1-5.
5. Alarslan E, Aygün T. Determination of some milk yield and reproduction characteristics of Kıvırcık sheep in Yalova. BSJ Agric. 2019; 2(2): 86-92.
6. Sahin Y, Olfaz M. Investigating the breeding and lamb growing practices applied by sheep farmers in Tokat region. BSJ Agric. 2019; 2(3): 171-180.

7. Ateş A, Çam MA. Structural features of sheep farms in Ordu province. BSJ Agric. 2022; 5(3): 323-328.
8. FAO. Faostat, Live Animals Data. Available online: http://www.fao.org/faostat/en/#data/QL (accessed on 20 October 2021).
9. Huby M, Griffon L, Moureaux S, De Rochambeau H, Danchin-Burge C, Verrier E. Genetic variability of six French meat sheep breeds in relation to their genetic management. Genet. Sel. Evol. 2003; 35: 637-655.
10. Vitezica ZG, Moreno CR, Bouix J, Barillet F, Perret G, Elsen M. A study on associations between PrP genotypes and meat traits in French sheep breeds. Anim Sci. 2005; 81(3): 325-330.
11. Vitezica ZG, Moreno CR, Bodin L, François D, Barillet F, Brunel JC, Elsen JM. No associations between PrP genotypes and reproduction traits in INRA 401 sheep1. J Anim Sci. 2006; 84: 1317-1322.
12. Sahin O, Aytekin I, Boztepe S, Keskin I, Karabacak A, Altay Y, Bayraktar M. Relationships between FAMACHA© scores and parasite incidence in sheep and goats. Trop Anim Health Prod. 2021; 53: 331.
13. Sabbioni A, Beretti V, Superchi P, Ablondi M. Body weight estimation from body measures in Cornigliese sheep breed. Ital J Anim Sci. 2020; 19(1): 25-30.
14. Ağyar O, Özköse E, Ekinci MS, Akyol İ. Investigation of live weight measurements of morkaraman lambs according to various times in terms of different variables. BSJ Agri. 2020; 3(3): 193-199.
15. Sakar ÇM, Ünal İ, Okuroğlu A, Coşkum Mİ, Zülkadir U. Prediction of live weight from chest girth from birth to 12 months of age in Yerli Kara cattle. BSJ Agric. 2020; 3: 200-204.
16. Khan MA, Tariq MM, Eyduran E, Tatliyer A, Rafeeq M, Abbas F, Rashid N, Awan MA, Javed K. Estimating body weight from several body measurements in Harnai sheep without multicollinearity problem. J Anim Plant Sci. 2014; 24(1): 120-126.
17. Ali M, Eyduran E, Tariq MM, Tirink C, Abbas F, Bajwa MA, Baloch MH, Nizamani AH, Waheed A, Awan MA, Shah SH, Ahmad Z, Jan S. Comparison of artificial neural network and decision tree algorithms used for predicting live weight at post weaning period from some biometrical characteristics in Harnai sheep. Pakistan J Zool. 2015; 47: 1579-1585.
18. Faraz A, Tirink C, Eyduran E, Waheed A, Tauqir NA, Nabeel MS, Tariq MM. Prediction of live body weight based on body measurements in Thalli sheep under

tropical conditions of Pakistan using CART and MARS. Trop Anim Health Prod. 2021; 53(2): 1-12.

19. Yavuz E, Şahin M. Investigation of Parametric, Non-Parametric and Semiparametric Methods in Regression Analysis. Sakarya Univ J Sci, 2022; 26(6): 1111-1116.
20. Mendes M, Akkartal E. Regression tree analysis for predicting slaughter weight in broilers. Ital J Anim Sci, 2009; 8: 615-624.
21. Sahin M, Yavuz E, Uckardes F. Multicollinearity Problem and Bias Estimates in Japanese Quail. Pakistan J Zool. 2018; 50(2): 757-761.
22. Tırınk C. Estimating of birth weight using placental characteristics in the presence of multicollinearity. BSJ Eng. Sci. 2020; 3: 138-145.
23. Yavuz E, Şahin M. Semiparametric regression models and applicability in agriculture. BSJ Agri. 2022; 5(2): 160-166.
24. Tyasi TL, Eyduran E, Celik S. Comparison of tree-based regression tree methods for predicting live body weight from morphological traits in Hy-line silver brown commercial layer and indigenous Potchefstroom Koekoek breeds raised in South Africa. Trop Anim Health Prod, 2021; 53: 7.
25. Kurnaz B, Önder H, Piwczynski D, Kolenda M, Sitkowska B. Determination of the best model to predict milk dry matter in high milk yielding dairy cattle. Acta Sci. Pol. Zootechnica, 2021; 20(3): 41-44.
26. Eyduran E, Zaborski D, Waheed A, Celik S, Karadas K, Grzesiak W. Comparison of the predictive capabilities of several data mining algorithms and multiple linear regression in the prediction of body weight by means of body measurements in the indigenous Beetal goat of Pakistan. Pakistan J Zool, 2017; 49(1): 257-265.
27. Eyduran E, Akin M, Eyduran SP. Application of Multivariate Adaptive Regression Splines through R Software; Nobel Academic Publishing; Ankara, Türkiye, 2019.
28. Olfaz M, Tirink C, Onder H. Use of CART and CHAID algorithms in Karayaka sheep breeding. J. Kafkas Univ. Vet. Fak. Derg. 2019; 25(1): 105-110.
29. Ricordeau G, Tchamitchian L, Brunel JC, Nguyen TC, François D. La race ovine INRA 401 : un exemple de souche synthétique. INRA Prod. Anim. 1992; 1992: 255-262.
30. Breiman L, Friedman JH, Olshen RA, Stone CJ. Classification and regression trees; Chapman and Hall Wadsworth Inc.; New York, NY, USA, 1984.
31. Friedman J. Multivariate adaptive regression splines. Ann Stat, 1991; 19(1): 1-67.

32. Akin M, Eyduran SP, Eyduran E, Reed BM. Analysis of macro nutrient related growth responses using multivariate adaptive regression splines. Plant Cell, Tissue Organ Cult. 2020; 140: 661-670.
33. Tırınk C, Piwczyński D, Kolenda M, Önder H. Estimation of body weight based on biometric measurements by using random forest regression, support vector regression and CART algorithms. Animals, 2023; 13: 798.
34. Coşkun G, Şahin Ö, Altay Y, Aytekin İ. Final fattening live weight prediction in Anatolian merinos lambs from some body characteristics at the initial of fattening by using some data mining algorithms. BSJ Agri. 2023; 6: 47-53.
35. Arthur CK, Temeng VA, Ziggah YY. Multivariate Adaptive Regression Splines (MARS) approach to blast-induced ground vibration prediction. Int. J. Min. Reclam, 2020; 34(3): 198-222.
36. Zaborski D, Ali M, Eyduran E, Grzesiak W, Tariq MM, Abbas F, Waheed A, Tirink C. Prediction of selected reproductive traits of indigenous Harnai sheep under the farm management system via various data mining algorithms. Pakistan J Zool, 2019; 51: 421-431.
37. Çanga D, Yavuz E, Efe E. Prediction of Egg Weight Using MARS data mining Algorithm through R. KSU J. Agric. Nat. 2021; 24(1): 242-251.
38. Çanga D. Use of MARS Data Mining algorithm based on training and test sets in determining carcass weight of cattle in different breeds. J. Agri. Sci. 2022; 28(2): 259-268.
39. Çanga D, Boğa M. Detection of correct pregnancy status in lactating dairy cattle using MARS data mining algorithm. Turk J. Vet. Anim. Sci. 2022; 46: 809-819.
40. Nguyen QT, Fouchereau R, Frénod E, Gerard C, Sincholle V. Comparison of forecast models of production of dairy cows combining animal and diet parameters. Comput. Electron. Agric. 2020; 170: 105258.
41. Smola AJ, Schölkopf BA. Tutorial on support vector regression. Stat. Comput. 2004; 14: 199-222.
42. Kavaklioglu K. Modeling and prediction of Turkey’s electricity consumption using Support Vector Regression. Appl. Energy, 2011; 88: 368-375.
43. Alonso J, Castañón ÁR, Bahamonde A. Support Vector Regression to predict carcass weight in beef cattle in advance of the slaughter. Comput. Electron. Agric. 2013; 91: 116-120.

44. Patel AK, Chatterjee S, Gorai AK. Development of a machine vision system using the support vector machine regression (SVR) algorithm for the online prediction of iron ore grades. IAPR Mach Vis Appl, 2017; 12: 197-210.
45. Grzesiak W, Zaborski D. Examples of the use of data mining methods in animal breeding. In: Data mining applications in engineering and medicine (ed. A Karahoca); InTech, Rijeka: Croatia, 2012.
46. Tatliyer A. The Effects of Raising Type on Performances of Some Data Mining Algorithms in Lambs. KSU J. Agric. Nat. 2020; 23(3): 772-780.
47. R Core Team. R: A language and environment for statistical computing. R Foundation for Statistical Computing, Vienna, Austria. 2022, URL: https://www.R-project.org/.
48. Revelle W. psych: Procedures for Personality and Psychological Research, Northwestern University, Evanston, Illinois, USA, 2022, https://CRAN.R-project.org/package=psych Version = 2.2.9.
49. Kuhn M. caret: Classification and Regression Training. R package version 6.0-93, 2022, URL: https://CRAN.R-project.org/package=caret.
50. Meyer D, Dimitriadou E, Hornik K, Weingessel A, Leisch F. e1071: Misc Functions of the Department of Statistics, Probability Theory Group (Formerly: E1071), TU Wien. R package version 1.7-12, 2022, URL: https://CRAN.R-project.org/package=e1071.
51. Eyduran E. ehaGoF: Calculates Goodness of Fit Statistics. R package version 0.1.1, 2020, URL: https://CRAN.R-project.org/package=ehaGoF.
52. Iqbal F, Waheed A, Faraz A. Comparing the Predictive Ability of Machine Learning Methods in Predicting the Live Body Weight of Beetal Goats of Pakistan. Pakistan J Zool, 2022; 54(1): 231-238.
53. Marco R, Ahmad SSS, Ahmad S. Bayesian hyperparameter optimisation and Ensemble Learning for Machine Learning Models on software effort estimation. Int. J. Adv. Comput. Sci. Appl. 2022; 13(3): 419-429.
54. Tırınk C. Comparison of Bayesian Regularized Neural Network, Random Forest Regression, Support Vector Regression and Multivariate Adaptive Regression Splines Algorithms to Predict Body Weight from Biometrical Measurements in Thalli Sheep. J. Kafkas Univ. Vet. Fak. Derg. 2022; 28(3): 411-419.
55. Kumar P, Kumar-Singh AA. Comparison between MLR, MARS, SVR and RF Techniques: Hydrological Time-series Modeling. J. Human Earth Future 2022; 3(1): 90-98.

56. Komadja GC, Rana A, Glodji LA, Anye V, Jadaun G, Onwualu PA, Sawmliana C. Assessing Ground Vibration Caused by Rock Blasting in Surface Mines Using Machine-Learning Approaches: A Comparison of CART, SVR and MARS. Sustainability 2022; 14: 11060.

 Click here to access/download;Figure;Figure 1.docx

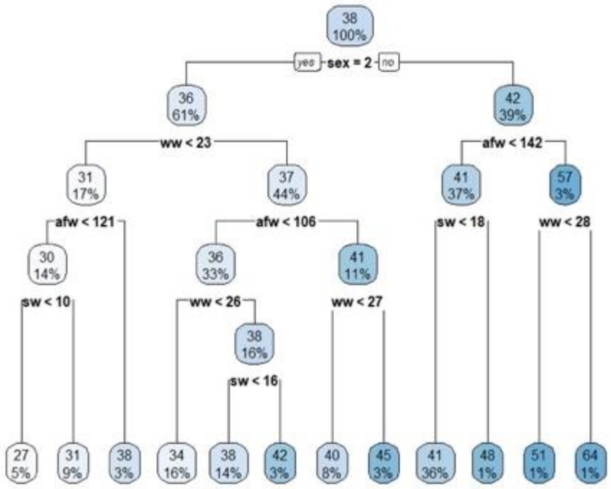

Figure 1. Constructed CART regression tree diagram.

Click here to access/download;Figure;Figure 2.pdf

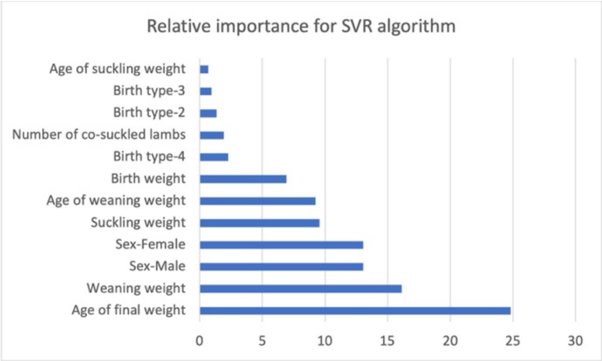

Figure 2. Sensitivity analysis for support vector regression algorithm
