# Supplementary material for: Comparison of the data mining and machine learning algorithms for predicting the final body weight for Romane sheep breed
Source: PLoS One. 2023 Aug 3;18(8):e0289348. doi: 10.1371/journal.pone.0289348 (PMC10399827; doi:10.1371/journal.pone.0289348)
Supplement: Attachment — Submitted filename: Response Letter.docx [file pone.0289348.s002.docx]

This document is a response letter on the manuscript ***#*** ***PONE-D-23-13209 entitled “Comparison of the Data Mining and Machine Learning Algorithms for Predicting the Final Body Weight for Romane Sheep Breed”.***

Dear Editor,

Many thanks for sharing valuable comments of you and reviewers with us on improving the manuscript PONE-D-23-13209 entitled “Comparison of the Data Mining and Machine Learning Algorithms for Predicting the Final Body Weight for Romane Sheep Breed”. I have given answers to all comments of the reviewers evaluating my manuscript using red color fonts. Also, red color font on the revised manuscript has been used for indicating all the corrections made by Reviewers.

With Best Regards

Assoc. Prof. Dr. Cem TIRINK

Corresponding author

**COMMENTS FOR THE AUTHOR**

**Reviewer #1:**

**Comments of Reviewer #1**

The aim of the manuscript is to estimate the optimal final body weight of Romane lambs from specific parameters (birth weight, suckling weight, age at suckling weight, weaning weight, age at weaning weight and age at final weight) using traditional statistical calculations and different data mining and machine learning algorithms (CART, MARS and SVR). This study will help sheep farmers to choose the right selection criteria and flock management, which is an important economic issue.

The strongest part of the manuscript is the statistical analysis, especially the textual evaluation of the results obtained. However, the introduction is very general. It is recommended that the problem statement be more specific.

Formally, the following numbered lines should be corrected:

46 Im-portance

204 Invalid table title

250 It is recommended to delete the letters 'h' from the table.

As there are so many abbreviations, it is recommended to summarise them in a list. It takes a lot of effort to find the abbreviations when reading the analysis.

It would also be useful to indicate the abbreviations in Figure 1 in the text (ww, sw, fw, etc.).

The use of units of measurement in tables is also recommended.

Overall, the manuscript is suitable for publication with the suggested changes.

**Response to Reviewer 1**

Thanks to reviewer 1 for the valuable comments. All corrections except the introduction were made according to the request of the reviewer 1. If the revision is not made in the introduction part, due to the unity of the text.

**Comment 2 of Reviewer #2**

Interesting article with adequate statistical analysis. Document writing deserves to be improved to a standard english.

Comments:

Lina 51: Update breeds references with more recent articles. Those published are between 2003 and 2006

Line 70: Sex (corret sex)

Line 80: Age of suckling weight: Two concepts, Weight and Age??

Suckling Weight or Weight at 30 days?

Line 85: age of weaning weight or weaning age?

Change on all document

Line 115: Add References 37,38 instead of 37, 38

Line 187: Objectify the term "Caret"

Line 195: Traits Use Units (kg) (days)

Line 278 - Discussion - Improve the english

Line 293, 309, 316, 324, 338, 346: Author aimed to compare?

Line 364 - Conclusion It should be more objective, especially in the opening paragraphs. At this point it discusses methodologies

**Response to reviewer 2**

Thanks to Reviewer 2 for the valuable comments. All corrections except some revisions were made according to the request of the reviewer 2.

**Comments Reviewer #3**

The revisions given by Reviewer 3 in the attachment have been taken into account.

**Response 3**

Thanks to Reviewer 3 for the valuable comments. All revisions were made according to the request of the reviewer 3.
