# Supplementary material for: Comparison of the data mining and machine learning algorithms for predicting the final body weight for Romane sheep breed
Source: PLoS One. 2023 Aug 3;18(8):e0289348. doi: 10.1371/journal.pone.0289348 (PMC10399827; doi:10.1371/journal.pone.0289348)
Supplement: Attachment — Submitted filename: Response Letter.docx [file pone.0289348.s003.docx]

This document is a response letter on the manuscript ***#*** ***PONE-D-23-13209R1 entitled “Comparison of the Data Mining and Machine Learning Algorithms for Predicting the Final Body Weight for Romane Sheep Breed”.***

Dear Editor,

Many thanks for sharing valuable comments of you and reviewers with us on improving the manuscript PONE-D-23-13209R1 entitled “Comparison of the Data Mining and Machine Learning Algorithms for Predicting the Final Body Weight for Romane Sheep Breed”. I have given answers to all comments of the reviewers evaluating my manuscript using red color fonts. Also, red color font on the revised manuscript has been used for indicating all the corrections made by Reviewers.

With Best Regards

Assoc. Prof. Dr. Cem TIRINK

Corresponding author

**COMMENTS FOR THE AUTHOR**

**Reviewer #1:**

**Comment #1**

Journal Requirements:

Please review your reference list to ensure that it is complete and correct. If you have cited papers that have been retracted, please include the rationale for doing so in the manuscript text, or remove these references and replace them with relevant current references. Any changes to the reference list should be mentioned in the rebuttal letter that accompanies your revised manuscript. If you need to cite a retracted article, indicate the article’s retracted status in the References list and also include a citation and full reference for the retraction notice.

**Response to Reviewer 1**

The citations in the manuscript were reviewed one by one. In addition, the places and requirements in the text were reviewed and it was determined that the citations in the text and in the references section were consistent.
